# The Relation of the Function of Vision to Health1Read before the Rochester Pathological Society, January 25, 1906

**Published:** 1906-05

**Authors:** Albert C. Snell

**Affiliations:** Rochester, N. Y.


### The Relation of the Function of Vision to Health.[1]

BY ALBERT C. SNELL, M. D., Rochester, N. Y.

IT has been said that most of our pathology has its origin in abnormal physiology. Medicine is full of examples of the truth of this statement, but it is my purpose in this paper to limit the discussion of this truth to a consideration of some of the pathological conditions other than those of the eye itself, with which the visual function is directly and indirectly concerned. Indirectly, there are many diseased conditions with the production of which sight is connected. In most of these cases the connection is remote and scarcely worth considering. Hence in our discussion we shall take up but one of the conditions which may be so caused, the most important—namely, scoliosis.

Hygienists and pediatrists have often called our attention to the malposition which school children assume while sitting at their desks, showing that such positions are etiological in the production of spinal curvature. In some school rooms the desks are so placed that the light shines directly into the eyes of the pupils; in others the light comes entirely from the right. Both of these conditions are bad. In the first case when the light pours directly into the eyes, the retina soon becomes exhausted, there is a bright glare on objects looked at, and distinct vision is impossible. In the second case, where the light comes from the right with right handed people (since we write from left to right), shadows from hand and pen or pencil cover the words being written—an annoying and confusing condition. When the light constantly comes from these improper directions it causes the pupils habitually to assume incorrect positions in their efforts to avoid the too great glare and these annoying shadows. Observe the pupils at their desks in any room where the light comes from these directions and you will see the children unconsciously screwing themselves into all sorts of contortions in order to see comfortably or to see at all. Whether in school or out of it a little attention to the right kind of light coming from the right directions is of prime importance, for many cases of incipient spinal curvature may thus have their origin in malpositions habitually assumed by these young patients in their efforts to see, even when they possess perfectly normal eyes.

---

1. Read before the Rochester Pathological Society, January 25, 1906

Spinal curvature may also be produced indirectly by the use of defective eyes. Children with high degrees of myopia, with high degrees of hypermetropia, and with astigmatism, are forced into harmful positions because they are compelled to bring their eyes near their books in order to see. Thus they acquire the habit of sitting in cramped, stooped positions, and by this habit produce in themselves round shoulders, crooked backs, shallow chests, and the consequences which follow therefrom.

The direct way in which abnormal conditions are associated with the exercise of the visual function is through the reflex system. All physiological reflex function is difficult of exact comprehension and understanding. Even more difficult is it to reduce the abnormal workings of our reflexes to anything like a science. In spite of the accumulated knowledge of to-day there still lurks a great deal of mystery in our sympathetic nervous system. This is particularly true of eye reflexes, since the eye is the only organ in the body of which mathematical exactness of construction is required of the organ itself. A too long or too short eyeball, or a lack of perfect spherical curvature impairs its usefulness, and compensation is required of other parts if clear vision is to be obtained. Besides, how marvelous is the accurate performance of the extremely difficult task imposed on the fourteen ocular muscles—the two ciliary muscles and the twelve extrinsic ocular muscles—all of which must work together in perfect harmony in order to produce a clear and instantaneous picture from any possible point of view!

The slightest imbalance or failure of perfect adjustment of these muscles produces blurred images or diplopia—conditions which nature abhors and resists with all her available force. If we consider simply the delicacy of the anatomy and the sensitiveness of the adjustment of these very complicated parts we might logically be led into thinking—as some indeed have been—that very slight deviations from the normal would produce profound and widespread disturbances. Thus, one author is lead to make this exaggerated statement: "This almost infinitesimal variant of 1|300 of an inch, the thickness of a sheet of paper, in eyeball measurements may throw the unfortunate possessor out of the struggle for existence—or it may render him a most pathetic sufferer."

The determination of eyeball measurements—in other words, finding the existing refractive and muscular condition of the eye—is a comparatively simple matter, and on this part of the work all ophthalmologists agree. But what degrees of variation from the mathematically normal eye are responsible for systemic disturbances is the rock on which the opinions of ophthalmologists split into extremes. Dr. Gould, of Philadelphia, holds one extreme. After reading articles which have recently come from his fertile pen, one might naturally be led to believe that most of the diseases to which flesh is heir are caused by eye reflexes. The list covers almost the entire field of the practice of medicine; and if it be true that the eye is a potent factor in the etiology of such a large percentage of diseases you see clearly how difficult it is to practise medicine conscientiously without being an ophthalmologist.

In a discussion of this subject a physician recently said: "I don't care about your controversies. What I want to get is the truth about this matter," which is the aim of us all, but difficult of attainment. Truth in medicine is similar to some of the less essential truths in religion—it depends largely on the point of view. Nevertheless the following facts seem to stand out conspicuously. All functions are interdependent. No function is entirely independent. The statement that the visual function is "pre-eminent" and "all-important" as compared with every other function is absurd. In biology, digestion and assimilation precede vision. The sense of feeling is developed earlier than that of sight. Reproduction, the object and end of all life, certainly cannot be secondary to the function of vision. But even should this claim of pre-eminence and all-importance be true, it does not follow, therefore, that all disturbed functions and diseased organs are likely to have their disorders originate in a perverted visual function.

However, among all the exaggerated claims in regard to the causation of disease and of abnormal conditions which are said to be caused by misuse or overuse of the visual function there is undoubtedly much truth. That constant and excessive ciliary muscle work does produce reflex disturbances is unquestionable. That it does so in every case is far from the truth. It seems to me that in the discussion of this subject, the relation of ametropia to health, we have largely lost sight of a very important phase of the question. We have failed to take into consideration the peculiar characteristics of the individual. In considering this relation it is impossible to classify the amount of reflex disturbance according to the amount of the existing refractive deformity. Simply because in certain cases we find a low, or medium, or a high degree of ametropia it does not therefore follow that we shall find a definite, relative amount of systemic disturbance.

It is not the low or the highest degrees of refractive errors which are necessarily likely to produce the greatest amount of reflex disturbance; neither is it the medium nor the highest degrees which necessarily produce the greatest or the least amount of disturbance. In addition to an otherwise normal general muscular tone or strength and a normally functionating nervous mechanism, the amount of disturbance which any given refractive error produces in a particular case depends largely on that individual's quality of endurance or the lack of it. Other conditions being equal, the degree of ametropia which produces profound and wide spread disturbance in one case has not the slightest effect on another. What we may designate as the equilibrium of health—that is, a proper balance of all contributing functions which maintain health—is normally very stable in some individuals and quite unstable in others, so that the amount of irritation, whether from ametropia or from other sources which may disturb this equilibrium of health, is a varying quantity with different individuals.

In considering, then, the relation of ametropia to reflex disturbances this individual equation should not be forgotten, as it not only determines the stability with which this equilibrium is maintained but it also largely determines the limit of systemic disturbance which ametropia may cause,—it is the measure of the individual's powers of endurance. Let us now consider under the topics, the circulatory, the digestive, and the nervous systems, the extent to which reflex irritation may be a factor in the etiology of disease or in disturbing normal functions.

The circulatory system is considered first because, according to certain theorists, this seems to be the important connecting link between "eye strain" and disease. It is claimed that an overtaxed visual organ may be "responsible for" and "capable of causing" a state of increased vasomotor irritability, which in turn leads to "an exaggerated or disordered vasomotor action." Cases of supposed vasodilation are reported to be cured by the relief of the existing ametropia. For example, in Dr. Gould's work* a case of dysmenorrhea caused by "exaggerated vasodilation of the internal genitalia was clearly shown to be due to "eye strain." It was clearly shown because the patient was relieved by wearing spectacles although an operation for the removal of her ovaries had been proposed. Following this same theory and using this same kind of logic, frontal sinus disease, pharyngitis, laryngitis, pneumonia, appendicitis, nephritis and almost all acute inflammations are shown to be due to ametropia.

In a word, according to this theory, the following is the roundabout way in which systemic disease is caused:

(a) Abnormal eyes cause abnormal reflex irritation to the vasomotor system.

(b) Abnormal reflex irritation to the vasomotor system causes vasomotor dilation.

(c) Vasomotor dilation causes disturbed circulation.

(d) Disturbed circulation causes perverted nutrition.

(e) Perverted nutrition makes fallow the ground for all sorts of diseases.

In regard to the influence of the visual function on the digestive system, it is a fact that a constant reflex disturbance produced by an overtaxed visual apparatus, due to hypermetropia or to astigmatism, can and does manifest itself by various disturbances of the digestive function. There is no doubt that nausea, and even vomiting are thus produced. Make for yourself the simple experiment of putting on a one or two diopter astigmatic lens and the majority of you will experience peculiar stomach sensations.

I believe that gastric neuralgia and nervous dyspepsia often have an intimate relation with abnormal eyes, and it is in these two classes of cases that the correction of ocular defects does most good. Examples of digestive disorders which have been cured by correcting refractive errors are common. The majority of such cases are of the nervous type. In passing let me say that digestive disturbances likewise produce asthenopia. There is no doubt that these two functions react on each other in a sort of vicious circle.

It is with the nervous system that the visual function is most intimately associated. But of the numerous nervous diseases there are three with which eye reflexes are commonly considered to be in some measure etiological—insanity, epilepsy, and chorea. But in my opinion eye reflexes have absolutely no causal relation to these diseases although they may possibly be an exciting factor in aggravating their symptoms.

With reference to insanity I can find no clinical evidence which would show that the use, abuse or defect of the visual function has ever been either the direct, or the remote cause of this disease, or that such use, or abuse or defect has aggravated the symptoms to any extent. But when patients afflicted with insanity, are suffering from any of the annoying symptoms produced by ocular errors they should have the same attention as the mentally balanced for the relief of such symptoms.

Concerning epilepsy some painstaking experiments have been made to determine whether or not the correction of refractive errors has any influence on epileptic seizures. So far, according to the majority report, these experiments indicate that the wearing of glasses has no effect either in decreasing the number of attacks or in the slightest degree modifying the seizures. However, I should like to report at this point one case in which the correction of an existing ametropia may have been of decided benefit.

Wm. B———, age 24, occupation— baker, was referred to me by Dr. Charles Reitz, December 22, 1900. He gave a history of having had several attacks of epileptic seizures two years previous to 1900, then of being free until November, 1900, when he had four rather severe seizures, being unconcious, biting his tongue, etc. From 1898 to 1900 inclusive was under bromides almost constantly. He gained weight while taking bromides. Patient very rarely has any headache, but says that when he reads at night "the type constantly fades away in front of his vision." Has never had glasses. Under homatropine mydriasis his refraction was found to be

O. D. plus 4.50 S. — plus 1.50 c. ax. 105°

O. S. plus 4.50 S. — plus 1.50 c. ax. 110°

with vision in O. D. 20|30, O. S. 20|20 partly. Glasses were ordered less 1.00 D. sph. for constant use. Since wearing glasses, December 22, 1900, patient was entirely free from seizures until December 1905. Patient changed his occupation about the time he began wearing glasses, taking up farming. During these five years 1900-1905 no bromides were taken. The seizure took place in December, 1905, and was a very slight one. Patient did not lose consciousness, did not fall nor injure himself. He was under intense excitement at the time. Another more severe attack (patient losing consciousness), occurred again in January, 1906. He was again under intense excitement. While out hunting he attempted to catch a rabbit alive as it came out of a hole in the ground. Just as the rabbit appeared he lost consciousness.

On February 7, 1906, I gave him within one quarter diopter of full correction and no more seizures have occurred. In this case the correction of ametropia apparently afforded relief, patient being entirely free from attack for five years. However, it takes a study of a large number of cases to establish a principle.

In chorea, also, much good has been claimed to come from the use of proper glasses. As far as the etiology is concerned, I believe there is no scientific evidence to prove that refractive errors are essentially causal. The essential etiological factors in these cases are more profound than an eye reflex. However, choreic children belong to the neurotic type and their nervous mechanism is more easily upset than that of perfectly healthy children; therefore every discoverable source of nervous irritation should be removed. Reflex disturbances produced by abnormal eyes are often a fruitful source for aggravating these sensitive nervous systems, and the patients derive much comfort from the proper glasses. I believe that the correction of refractive errors has a decidedly favorable influence on this condition.

Despite the fact that eye reflexes are not directly etiological in the production of nervous diseases they are most intimately and directly related to the functional neurosis. When there is an unstable nervous equilibrium there is no doubt that the abnormal amount of work which oversighted eyes require, and the disturbance to the reflex nervous system which is produced by astigmatism, are potent factors in bringing on nervous exhaustion. In such cases because there is a sensitive, high-strung, unstable, nervous mechanism, an abnormal visual function is an essential agent, being often the last straw which brings down the balance at the wrong end. The stress of overwork, the burden of worry are usually the principal factors in bringing the nervous system to the tension—breaking point; and when there is added to these the constant irritation of ametropia, the crisis is reached and a breakdown occurs. That the correction of ametropia does accomplish much in restoring these patients to useful lives, there can be no doubt. I believe that among these patients even the lower degrees of refractive errors should be corrected since such patients magnify even slight abnormalities. The relief which the correction of ametropia affords does much to relieve the entire nervous system.

Headache, in its relation to the eye, has been discussed so often that I shall give this topic only passing notice. With reference to headaches in general it may be said that probably there is no single known affliction which causes a greater sum total of suffering or more loss of time. Scarcely any individual is free from it. It manifests itself in connection with such a great number and variety of diseased conditions and disturbed functions that it is not to be wondered at that there is difficulty in locating the cause of the disturbance. Of an eye headache in particular, it may be said, that there is no special characteristic. Such headaches vary from a slight sense of pressure in the head to actual prostration associated with nausea and vomiting. The eye headache may be located in almost any part of the head. Most often it is frontal. It may be supraorbital, between the eyes, back of the eyes, temporal or occipital; but its location does not distinguish it from other forms of headache. This complaint is the one which the ophthalmologist hears most often. Yet it is only by careful examination of the existing ocular condition that he can decide whether or not the eye is the probable source of this reflex disturbance. When headaches are ocular in origin the relief afforded by the correction of the ametropia is positive and gratifying.

In health one should be able to enjoy life performing all functions without distressing symptoms of any kind. It follows then, that in health the eyes should perform their function without discomfort either to themselves or to any other part of the body.

This is not merely theory—one could cite many examples of strong, well people who have had high degrees of ametropia through a large part of their lives and who never had a headache or any other reflex disturbance. An unhealthy body has much more influence on the visual function than does an abnormal eye on a healthy body.

I believe that it usually requires some other element or elements than simply an eye reflex to produce distressing symptoms either in the eye itself or in more remote parts. There must be in addition to the eye reflex some individual characteristic, hereditary predisposition, or lack of vitality which renders the system unable to perform its functions without friction. However, if for any reason, whether from constitutional weakness, heredity, the enervating influence of our modern civilization, or actual disease, the system is weakened in any part, or thrown out of balance, then it is that an eye reflex does play an important role, and without doubt contributes much to human suffering. When we are dealing with any of these conditions the correction of existing ametropia is of prime importance and is a valuable means of enabling us to relieve many distressing symptoms or to restore health.

It has been my desire in this paper to emphasize the fact that the eye is only a part of the body, and that its influence in relation to health is not of greater importance than that of other and more vital organs, and lastly that an ophthalmologist who fails to take into consideration the relations of all bodily functions, their interdependence one on another, does not do his full duty to his patient.

**Biographic Clinics, Vol. III.*

53 SOUTH FITZHUGH STREET.

